# Synergistic effects of soil and foliar selenium application on growth, yield, and nutritional biofortification of okra (*Abelmoschus esculentus* L.) under open-field conditions

**DOI:** 10.3389/fpls.2026.1918333

**Published:** 2026-09-16

**Authors:** Gomathi Arivalagan, Utpal Das

**Affiliations:** Department of Horticulture and Food Science, VIT School of Agricultural Innovations and Advanced Learning, Vellore Institute of Technology, Vellore, Tamil Nadu, India

**Keywords:** biofortification, growth parameters, okra, selenium, sustainable agriculture

## Abstract

Selenium (Se) is an essential trace element known to enhance plant growth, antioxidant metabolism, stress tolerance, and nutritional quality, making it an important component of sustainable agronomic biofortification strategies. The present study evaluated the synergistic effects of soil and foliar selenium application on the growth, yield, biochemical composition, and mineral enrichment of okra (*Abelmoschus esculentus* L.) under open-field conditions at the Sevoor farm of Vellore Institute of Technology. The experiment was conducted using a Randomized Block Design comprising seven treatments with three replications. Selenium was applied through soil and foliar methods at different concentrations, and growth, yield, biochemical, and mineral parameters were assessed. Significant treatment effects were observed (p ≤ 0.05). Among the treatments, combined soil and foliar application of selenium at 25 ppm (T_5_) showed significant effects, recording the highest plant height (139.92 cm), branches (30 plant^-1^), flowers (60 plant^-1^), pods (80 plant^-1^), and yield (760 g plant^-1^). Treatment T_5_ also significantly enhanced chlorophyll, carotenoids, carbohydrates, proteins, phenolics, flavonoids, antioxidant activity, and essential mineral accumulation compared to the untreated control. These findings demonstrate that optimized selenium treatments through integrated soil and foliar application can improve crop productivity, nutritional quality, and sustainability, supporting climate-resilient and nutritionally improved agricultural systems for future food security.

## 1 Introduction

Selenium (Se) is an essential micronutrient for humans, and its significant role in disease prevention, immune regulation, and reduction of cancer risk has been extensively documented (Rayman, 2012). Deficiency of this trace element, often referred to as “hidden hunger,” is widespread in many regions of the world due to inadequate dietary Se intake and low Se availability in agricultural soils (Kumar et al., 2016). In recent years, selenium biofortification has emerged as a sustainable and effective strategy to improve the Se concentration of edible crop parts, thereby enhancing human nutrition through daily dietary intake (Bañuelos et al., 2017; Rayman, 2020). Agronomic biofortification not only addresses micronutrient malnutrition but also contributes to sustainable agriculture by improving crop productivity, nutritional quality, and resilience under changing environmental conditions (Ros et al., 2016; Hasanuzzaman et al., 2020). Food crops, particularly cereals and vegetables, are considered suitable targets for Se biofortification because of their regular consumption and potential contribution to human dietary requirements (Poblaciones et al., 2014; Schiavon and Pilon-Smits, 2017). Selenium-enriched vegetables possess enhanced antioxidant activity, improved nutritional quality, and increased resistance to environmental stresses, thereby offering additional health benefits to consumers (Abbas et al., 2022). Furthermore, selenium plays a beneficial role in plants by regulating antioxidant metabolism, photosynthesis, nutrient uptake, and stress tolerance under adverse environmental conditions such as drought, salinity, and temperature extremes (Feng et al., 2013; Liu et al., 2022). Hence, selenium-based biofortification represents a promising approach toward climate-resilient and nutritionally secure food production systems.

Okra (*Abelmoschus esculentus* L.) is an economically important vegetable widely cultivated in tropical and subtropical regions. It is highly valued for its rich nutritional composition, including vitamins, minerals, dietary fiber, and bioactive compounds such as polyphenols and flavonoids. Due to its broad adaptability, short growth duration, and high consumer acceptance, okra serves as an ideal candidate for selenium biofortification. Previous studies have demonstrated that selenium application can improve plant growth, yield attributes, antioxidant activity, and biochemical composition in okra under both normal and stress conditions (Sonali et al., 2024; Ali et al., 2025). Therefore, the development of Se-enriched okra may provide an effective dietary strategy to alleviate selenium deficiency in vulnerable populations while simultaneously supporting sustainable vegetable production. Currently, the major agronomic approaches for Se biofortification involve soil and foliar application of inorganic selenium fertilizers (Ros et al., 2016). Among these methods, foliar application is often considered more efficient because it minimizes selenium fixation and leaching losses in soil, thereby enhancing Se availability and uptake by plants (Zawislanski et al., 2003). Significant increases in selenium accumulation have been reported in cereals, vegetables, and pods following foliar Se application (Kápolna et al., 2012; Pezzarossa et al., 2012). The two commonly used inorganic forms of selenium fertilizers are selenate and selenite, whose uptake and translocation differ considerably among crops. Studies in rice and maize have shown that foliar application of selenate results in greater selenium accumulation compared with selenite treatments (Boldrin et al., 2013). In addition, the efficiency of Se uptake is influenced by the stage of application, with later growth-stage foliar treatments improving Se translocation and distribution in edible plant parts (Deng et al., 2017; Li et al., 2025).

Despite several studies on selenium biofortification, limited information is available regarding the combined effects of soil and foliar selenium application on growth performance, yield, and nutritional enhancement of okra under open-field conditions. Therefore, the present study was undertaken to evaluate the synergistic effects of soil and foliar selenium application at 20 and 30 days after sowing (DAS) on the growth, yield, and nutritional biofortification of okra. The findings of this study may contribute to the development of sustainable agronomic practices for producing nutrient-enriched vegetables capable of supporting future food and nutritional security.

## 2 Materials and methods

### 2.1 Experimental site and plant material

The field experiment was conducted during 2024–2025 at the Sevoor experimental farm of Vellore Institute of Technology, Tamil Nadu, India (12.97° N latitude and 79.19° E longitude). The region receives an average annual rainfall of approximately 1000–1030 mm and experiences a tropical semi-arid climate. The initial soil physicochemical properties were determined using standard soil analytical procedures described in the *Soil Analysis Handbook of Reference Methods* by Jones, 2018 and for soil selenium content (Tolu et al., 2011). The experimental soil was clay-loam in texture with pH ranging from 7.5 to 8.8. The initial soil nutrient status included available nitrogen (2.6 mg kg^-1^), Olsen phosphorus (22 mg kg^-1^), available potassium (186 mg kg^-1^), organic matter (14.1–31.7 g kg^-1^), and available selenium (0.01 mg kg^-1^). Okra seeds (*Abelmoschus esculentus* L. cv. NS7774) were procured from Namdhari Seeds Private Limited.

### 2.2 Experimental design and selenium application

The experiment was arranged in a Randomized Block Design (RBD) with seven treatments and three replications. Selenium was applied through soil and foliar application methods using selenium solutions prepared at concentrations of 25 and 50 ppm. The treatments consisted of soil application at 25 ppm (T_1_), soil application at 50 ppm (T_2_), foliar application at 25 ppm (T_3_), foliar application at 50 ppm (T_4_), combined soil and foliar application at 25 ppm (T_5_), combined soil and foliar application at 50 ppm (T_6_), and untreated control sprayed with deionized water (T_7_). Sodium selenite (Na_2_SeO_3_) was used as the selenium source. Soil application was performed at 20 days after sowing (DAS), whereas foliar application was carried out at 30 DAS. A total volume of 2 L of selenium solution was used separately for soil and foliar applications per plot (15 m²). Deionized water was used as the foliar control. The crop was maintained at a spacing of 60 × 30 cm. Farmyard manure (FYM) and neem cake were incorporated as basal organic nutrient sources, whereas no chemical fertilizers were applied during the experimental period. Irrigation and intercultural operations were carried out uniformly for all treatments following standard agronomic practices. The first harvest was initiated 45 days after sowing and repeatedly at the commercial pod stage, resulting in approximately 10–12 harvests with crop duration of 90 days. Pod yield was recorded separately at each harvest by weighing the freshly harvested pods from individual plants, and the cumulative yield across all harvests was calculated for each plant.

### 2.3 Growth and yield parameters

Germination percentage was recorded before the application of selenium treatments (at 19^th^ day). The plant height and branch number were monitored periodically during crop growth, only the final observations recorded at harvest were considered for statistical analysis and are presented in this study. Reproductive parameters such as number of flowers, number of pods, pod length, pod weight, and total yield per plant were recorded at harvest. These parameters were selected to assess the influence of selenium on reproductive efficiency, biomass allocation, and yield potential. Similar growth evaluation methods have been widely adopted in selenium biofortification studies in vegetable crops (Francini et al., 2023; Gui et al., 2021).

### 2.4 Biochemical analysis

#### 2.4.1 Chlorophyll and carotenoid content

Photosynthetic pigments were estimated following the acetone extraction method described by Lichtenthaler and Buschmann (2001). Fresh leaf tissue (0.1 g) was homogenized in 10 mL of 80% acetone and centrifuged at 5000 rpm for 10 min. The absorbance of the supernatant was recorded at 663, 645, and 470 nm using a UV–Visible spectrophotometer. Chlorophyll a, chlorophyll b, and total carotenoid contents were calculated using standard equations and expressed as mg g^-1^ fresh weight.

#### 2.4.2 Total soluble protein

Total soluble protein content was determined according to the method of Lowry et al. (1951). Fresh plant tissue (0.1 g) was homogenized in phosphate buffer and centrifuged to obtain the supernatant. An aliquot (1 mL) of the extract was mixed with alkaline copper reagent and incubated for 10 min at room temperature. Subsequently, Folin–Ciocalteu reagent was added, and absorbance was measured at 660 nm. Bovine serum albumin (BSA) was used as the standard for protein quantification.

#### 2.4.3 Total phenolic content

Total phenolic content was estimated using the Folin–Ciocalteu colorimetric method following Singleton et al. (1999). Methanolic extract (0.5 mL) was mixed with Folin–Ciocalteu reagent, distilled water, and 20% sodium carbonate solution. The reaction mixture was incubated at room temperature for 30 min, and absorbance was recorded at 700 nm. Results were expressed as mg gallic acid equivalent (GAE) g^-1^ fresh weight.

#### 2.4.4 Total flavonoid content

Total flavonoid content was determined using the aluminum chloride colorimetric method described by Ordóñez et al. (2006). Methanolic extract was mixed with aluminum chloride, sodium acetate, methanol, and distilled water and incubated for 30 min at room temperature. Absorbance was measured at 415 nm, and flavonoid concentration was expressed as mg quercetin equivalent (QE) g^-1^ fresh weight.

#### 2.4.5 Total antioxidant activity

Total antioxidant activity was determined using the Ferric Reducing Antioxidant Power (FRAP) assay according to Benzie and Strain (1999). The FRAP reagent was prepared by mixing acetate buffer, 2,4,6-Tris(2-pyridyl)-s-triazine (TPTZ) solution, and ferric chloride solution. Methanolic extract (0.5 mL) was mixed with FRAP reagent and incubated for 30 min in dark conditions. Absorbance was measured at 593 nm using a UV–Visible spectrophotometer. Antioxidant activity was quantified using an ascorbic acid standard curve.

### 2.5 Mineral and selenium analysis

Mineral analysis was performed using a PerkinElmer NexION 1000 ICP-MS following acid digestion of dried okra pod samples. The composite samples pooled from all field replications for each treatment. Approximately 0.5 g of oven-dried plant material was digested using concentrated nitric acid and hydrogen peroxide (Raina et al., 2020; Francini et al., 2023). Elemental quantification was done based on the measured ion signal intensity/counts per second (cps) and calibration curves by monitoring isotope78 Se and other elemental isotopes.

### 2.6 Statistical analysis

All data were analyzed using R Studio with R software (version 4.2.1, R Core Team, 2022). Analysis of variance (ANOVA) was performed, and treatment means were compared using Fisher’s Least Significant Difference (LSD) test. Differences were considered statistically significant at p ≤ 0.05.

## 3 Results and discussion

### 3.1 Growth and yield attributes

Selenium treatments significantly influenced the growth and yield performance of okra under open-field conditions (**Table 1**; **Figures 1**–**3**). Selenium has been reported to stimulate and enhance early growth by improving antioxidant defense mechanisms and membrane stability, thereby protecting seedlings from oxidative damage during establishment (Hasanuzzaman et al., 2020; Naseem et al., 2021).

**Table 1:** Effects of soil, foliar, and combined soil + foliar selenium applications on growth and yield of okra (*Abelmoschus esculentus* L.) under open-field conditions.

Treatment	Germination (%)	Plant height (cm)	No. of branches	No. of flowers	No. of pods	Pod weight (g)	Pod length (cm)	Yield (g plant^-1^)
T_1_	93.03^a^	110.03^bc^	21.03^d^	44.97^cd^	64.97^bc^	6.50^de^	16.50^ab^	422.5^c^
T_2_	85.00^a^	125.00^ab^	19.00^d^	42.97^cd^	60.00^cd^	6.83^d^	14.87^b^	408.0^c^
T_3_	80.00^a^	100.00^cd^	25.00^bc^	55.00^ab^	77.03^a^	9.30^ab^	18.60^a^	716.1^a^
T_4_	81.00^a^	95.00^cd^	22.00^cd^	50.00^bc^	66.00^bc^	8.00^c^	17.60^a^	528.0^b^
T_5_	92.00^a^	139.97^a^	30.00^a^	60.00^a^	80.00^a^	9.50^a^	19.00^a^	760.0^a^
T_6_	85.00^a^	130.00^a^	27.00^ab^	58.00^a^	71.00^ab^	8.20^bc^	18.30^a^	582.2^b^
T_7_	90.00^a^	90.00^d^	20.00^d^	40.00^d^	50.00^d^	5.50^e^	14.00^b^	319.2^d^
SEm ±	4.517	5.895	1.078	2.369	3.254	0.365	0.823	24.140
SEd ±	6.389	8.337	1.524	3.350	4.602	0.516	1.164	34.139
LSD/CD (5%)	13.919	18.164	3.321	7.299	10.026	1.125	2.537	74.382
CV (%)	9.04	9.05	7.97	8.18	8.41	8.22	8.40	7.93
Significance	NS	***	***	***	***	***	**	***

Values followed by different superscript letters within a column differ significantly at p ≤ 0.05.

NS, Non- Significant; **Significant at p ≤ 0.01; ***Significant at p ≤ 0.001.

**Figure 1:**
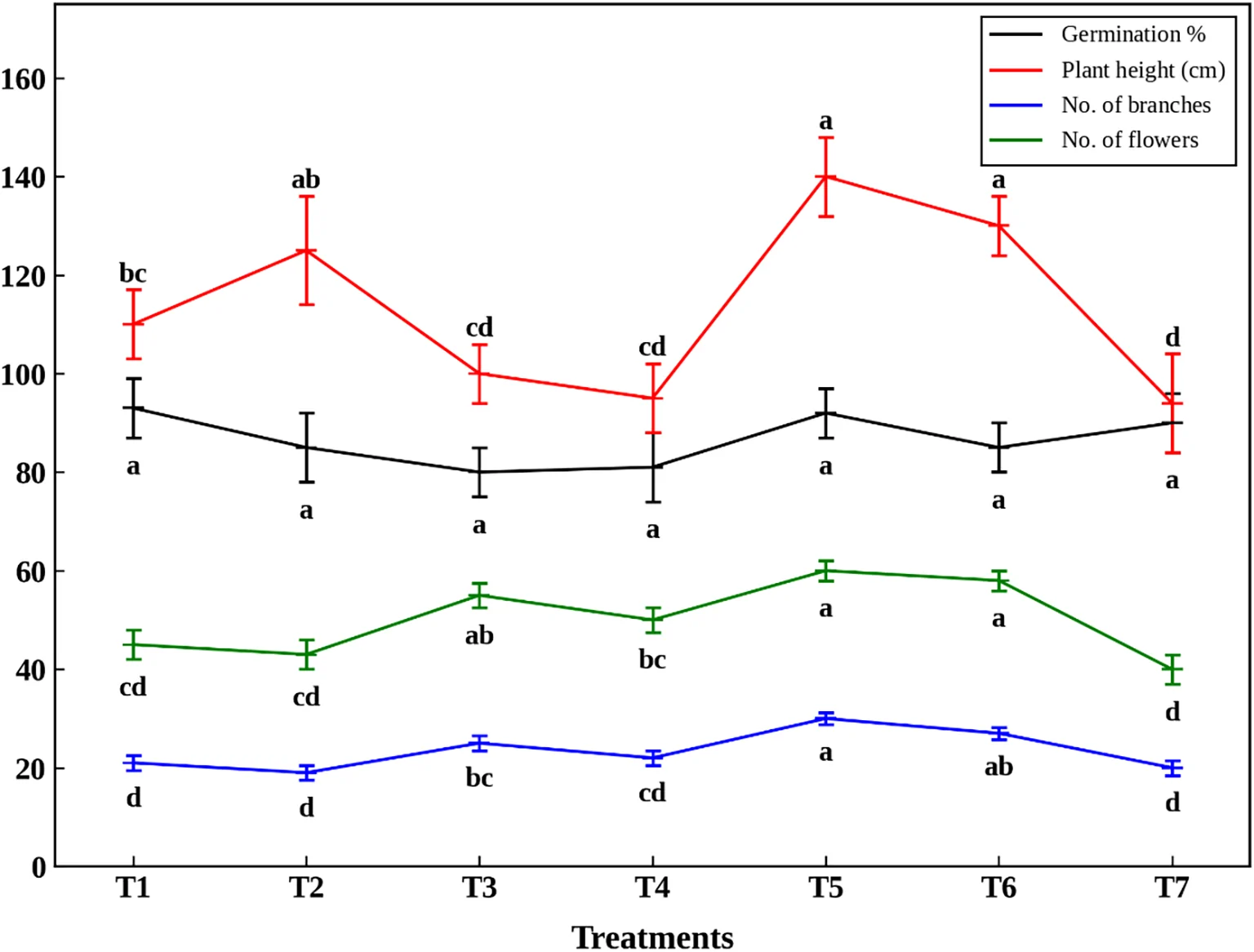
Effects of soil, foliar, and combined soil + foliar selenium application on germination percentage, plant height, branch number, and flower number of okra (*Abelmoschus esculentus* L.). T_1_, soil application (25 ppm Se); T_2_, soil application (50 ppm Se); T_3_, foliar application (25 ppm Se); T_4_, foliar application (50 ppm Se); T_5_, combined soil + foliar application (25 ppm Se); T_6_, combined soil + foliar application (50 ppm Se); T_7_, untreated control (deionized water). Values are expressed as mean ± standard error (SE) (n = 3). Different lowercase letters indicate significant differences among treatments according to Fisher’s least significant difference (LSD) test (*P* ≤ 0.05).

**Figure 2:**
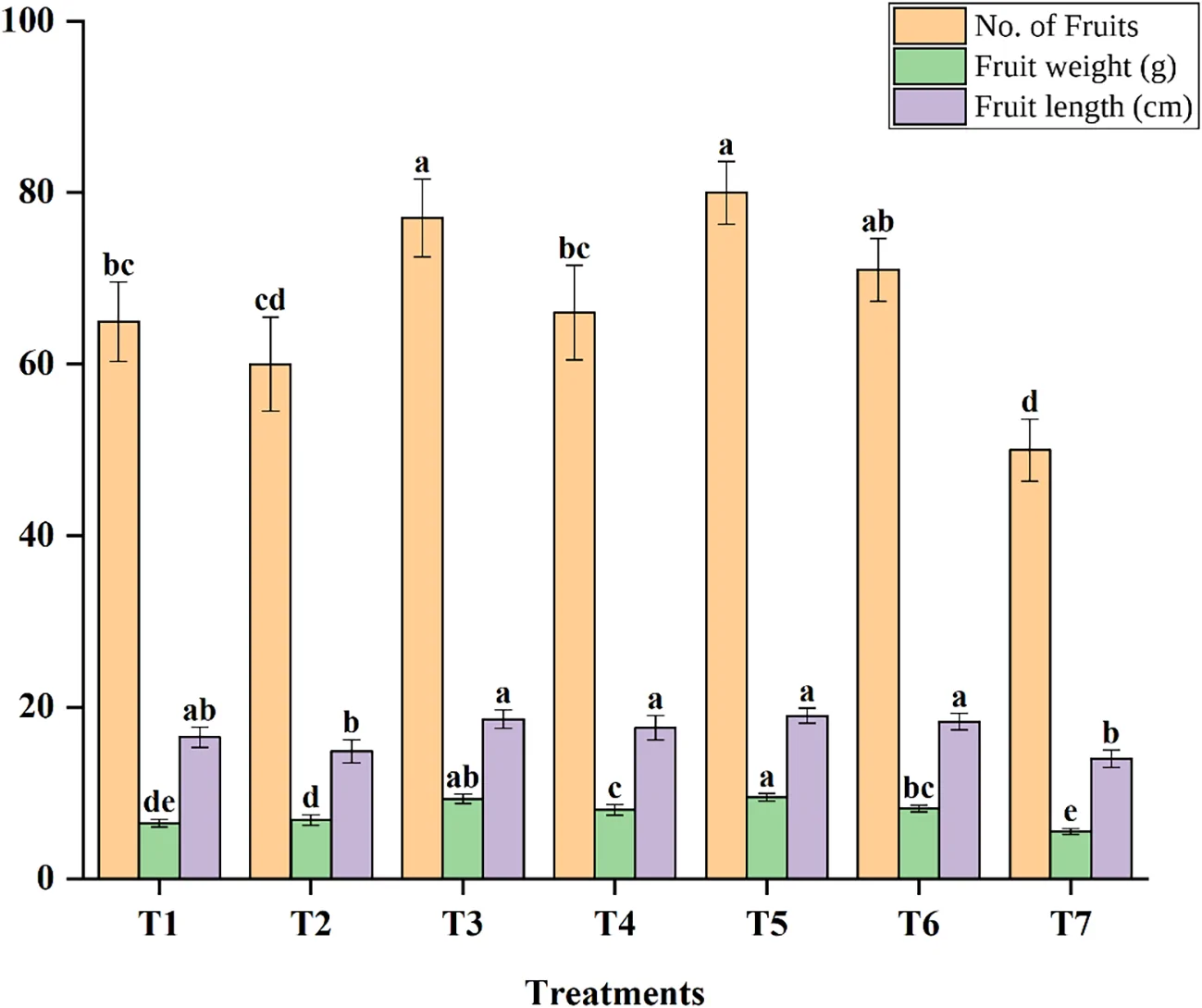
Effects of soil, foliar, and combined soil + foliar selenium application on number of fruits, fruit weight, fruit length, and yield per plant of okra (*Abelmoschus esculentus* L.). T_1_, soil application (25 ppm Se); T_2_, soil application (50 ppm Se); T_3_, foliar application (25 ppm Se); T_4_, foliar application (50 ppm Se); T_5_, combined soil + foliar application (25 ppm Se); T_6_, combined soil + foliar application (50 ppm Se); T_7_, untreated control (deionized water). Values are presented as mean ± standard error (SE) (n = 3). Different lowercase letters above the bars indicate significant differences among treatments according to Fisher’s least significant difference (LSD) test at *P* ≤ 0.05.

**Figure 3:**
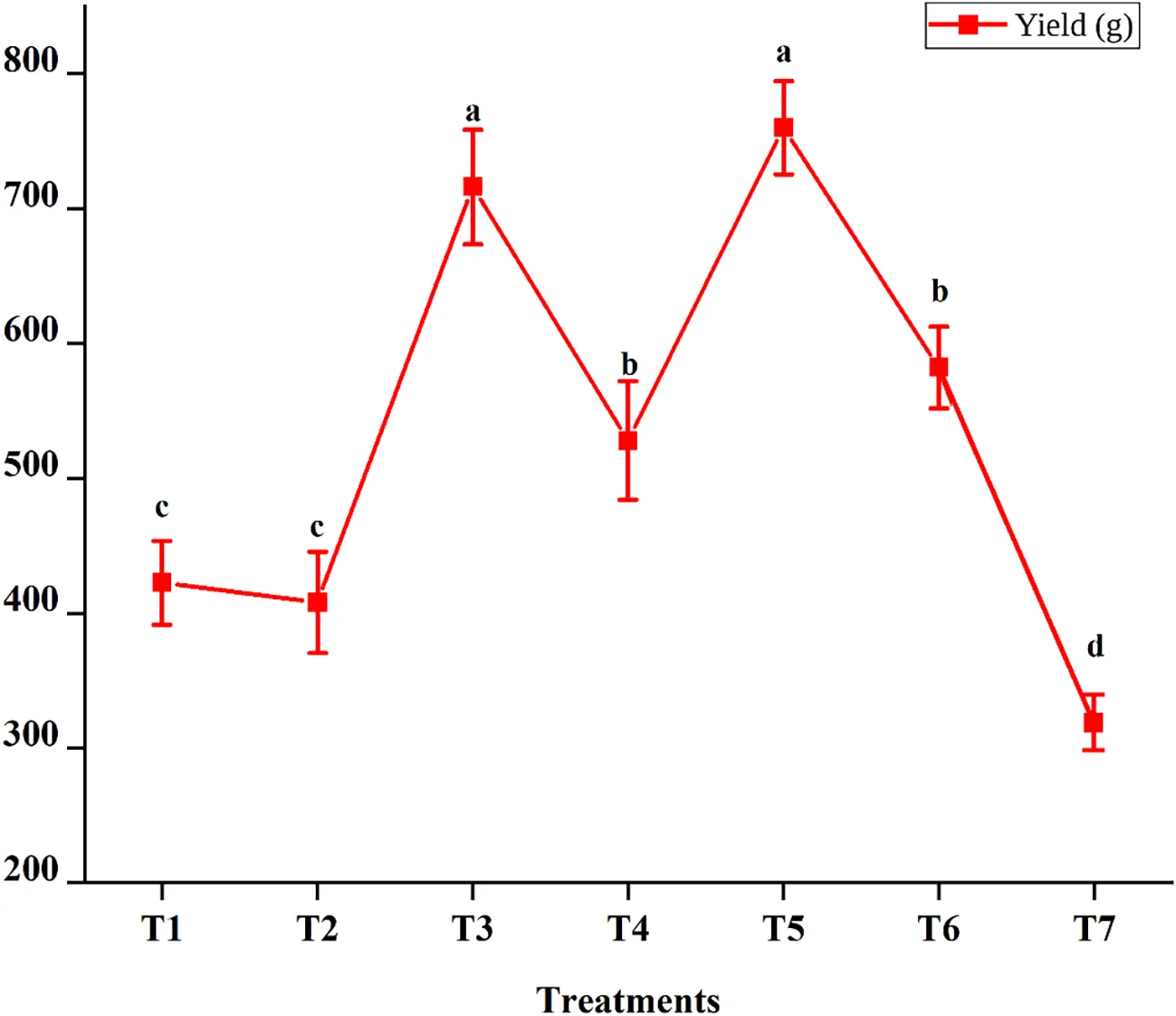
Effects of soil, foliar, and combined soil + foliar selenium application on yield per plant of okra (*Abelmoschus esculentus* L.). T_1_, soil application (25 ppm Se); T_2_, soil application (50 ppm Se); T_3_, foliar application (25 ppm Se); T_4_, foliar application (50 ppm Se); T_5_, combined soil + foliar application (25 ppm Se); T_6_, combined soil + foliar application (50 ppm Se); T_7_, untreated control (deionized water). Values are presented as mean ± standard error (SE) (n = 3). Different lowercase letters above the bars indicate significant differences among treatments according to Fisher’s least significant difference (LSD)test at *P* ≤ 0.05.

Significant improvements in vegetative and reproductive parameters were observed among the treatments. Treatment T_5_ recorded the highest plant height (139.97cm), number of branches (30 plant^-1^), flowers (60 plant^-1^), and pods (80 plant^-1^), which were significantly higher than those of the control treatment. Pod weight (9.50 g), pod length (19.0 cm), and yield (760 g plant^-1^) were also maximized under T_5_, representing an approximately 58% increase in yield over the untreated control (319.2 g plant^-1^). The enhanced growth and productivity under selenium treatments may be attributed to improved photosynthetic efficiency, hormonal regulation, nutrient assimilation, and enhanced enzymatic activity involved in primary metabolism (Pilon-Smits et al., 2009; Liao et al., 2025; Li H et al., 2025).

The significant effects of T_5_ compared with individual soil, foliar applications suggests that combined selenium delivery ensures both sustained root absorption and rapid foliar uptake, thereby improving selenium bioavailability throughout crop development. Similar findings have been reported in cucumber, lettuce, broccoli, and rice, where integrated selenium fertilization significantly improved vegetative growth, flowering, fruit set (pod set), and crop productivity (Boldrin et al., 2013; Francini et al., 2023; Gui et al., 2021). Selenium-mediated enhancement of yield has also been associated with improved pollen viability, better translocation of assimilates, and delayed senescence, ultimately contributing to increased reproductive efficiency (Zhang et al., 2014; Tao et al., 2026).

Furthermore, selenium is recognized as a beneficial element that enhances crop resilience under environmental stress conditions by regulating reactive oxygen species (ROS) detoxification pathways and improving stress-responsive metabolic functions (Liu et al., 2022). Such responses are particularly relevant for sustainable agriculture and climate-resilient crop production systems aimed at ensuring food security under changing environmental conditions. However, the comparatively lower performance observed under certain selenium treatments, particularly soil-applied treatments (T_1_ and T_2_), suggests that the response to selenium depended on the method and concentration of application. Although combined soil and foliar application at 25 ppm (T_5_) produced the best overall performance, the reduced response under higher soil-applied selenium concentrations may indicate concentration-dependent effects associated with metabolic toxicity or nutrient imbalance, as reported previously in rice and other crop species (Liu et al., 2024).

### 3.2 Biochemical parameters

Biochemical analyses revealed that selenium application significantly enhanced the accumulation of photosynthetic pigments, primary metabolites, and antioxidant compounds in okra plants (**Table 2**; **Figures 4**, **5**). Treatment T_5_ recorded the highest chlorophyll a (15.47 mg g^-1^), chlorophyll b (6.73 mg g^-1^), and carotenoid content (25.18 mg g^-1^), indicating improved chloroplast stability and photosynthetic activity under moderate selenium treatments. Selenium has been reported to protect chloroplast membranes from oxidative degradation and maintain the structural integrity of photosynthetic pigments under both normal and stress conditions (Karimi et al., 2020; Liu et al., 2022).

**Table 2:** Effects of soil, foliar, and combined soil + foliar selenium applications on the biochemical parameters of okra (*Abelmoschus esculentus* L.) under open-field conditions.

Treatment	Chlorophyll a (mg g^-1^)	Chlorophyll b (mg g^-1^)	Carotenoids (mg g^-1^)	Total carbohydrate (mg g^-1^)	Total phenol (mg g^-1^)	Total flavonoid (mg g^-1^)	Total antioxidant (mg g^-1^)	Total protein (mg g^-1^)
T_1_	5.37^c^	4.83^c^	10.58^d^	49.47^b^	66.43^a^	16.43^d^	19.97^c^	33.31
T_2_	12.77^b^	6.30^b^	22.44^ab^	41.23^c^	29.83^c^	16.43^d^	25.00^b^	28.48
T_3_	14.03^ab^	7.47^a^	24.26^ab^	53.30^b^	30.10^c^	31.57^a^	21.07^c^	26.57
T_4_	1.83^d^	1.10^d^	9.61^d^	48.13^bc^	23.77^cd^	17.77^d^	18.60^c^	34.36
T_5_	15.47^a^	6.73^ab^	25.18^a^	68.27^a^	59.83^a^	27.77^b^	31.60^a^	47.07
T_6_	12.97^b^	5.97^b^	22.01^b^	66.77^a^	39.80^b^	22.43^c^	29.70^a^	40.41
T_7_	2.07^d^	1.17^d^	17.41^c^	32.63^d^	22.10^d^	15.27^d^	17.60^c^	16.91
SEm ±	0.563	0.302	0.965	2.383	2.212	0.958	1.159	3.700
SEd ±	0.797	0.428	1.364	3.370	3.128	1.354	1.639	1.243
LSD/CD (5%)	1.736	0.932	2.972	7.342	6.815	2.951	3.611	10.470
CV (%)	10.59	10.92	8.89	8.03	9.86	7.86	8.59	13.06
Significance	***	***	***	***	***	***	***	***

Values followed by different superscript letters within a column differ significantly at p ≤ 0.05.

NS, Non-Significant; **Significant at p ≤ 0.01; ***Significant at p ≤ 0.001.

**Figure 4:**
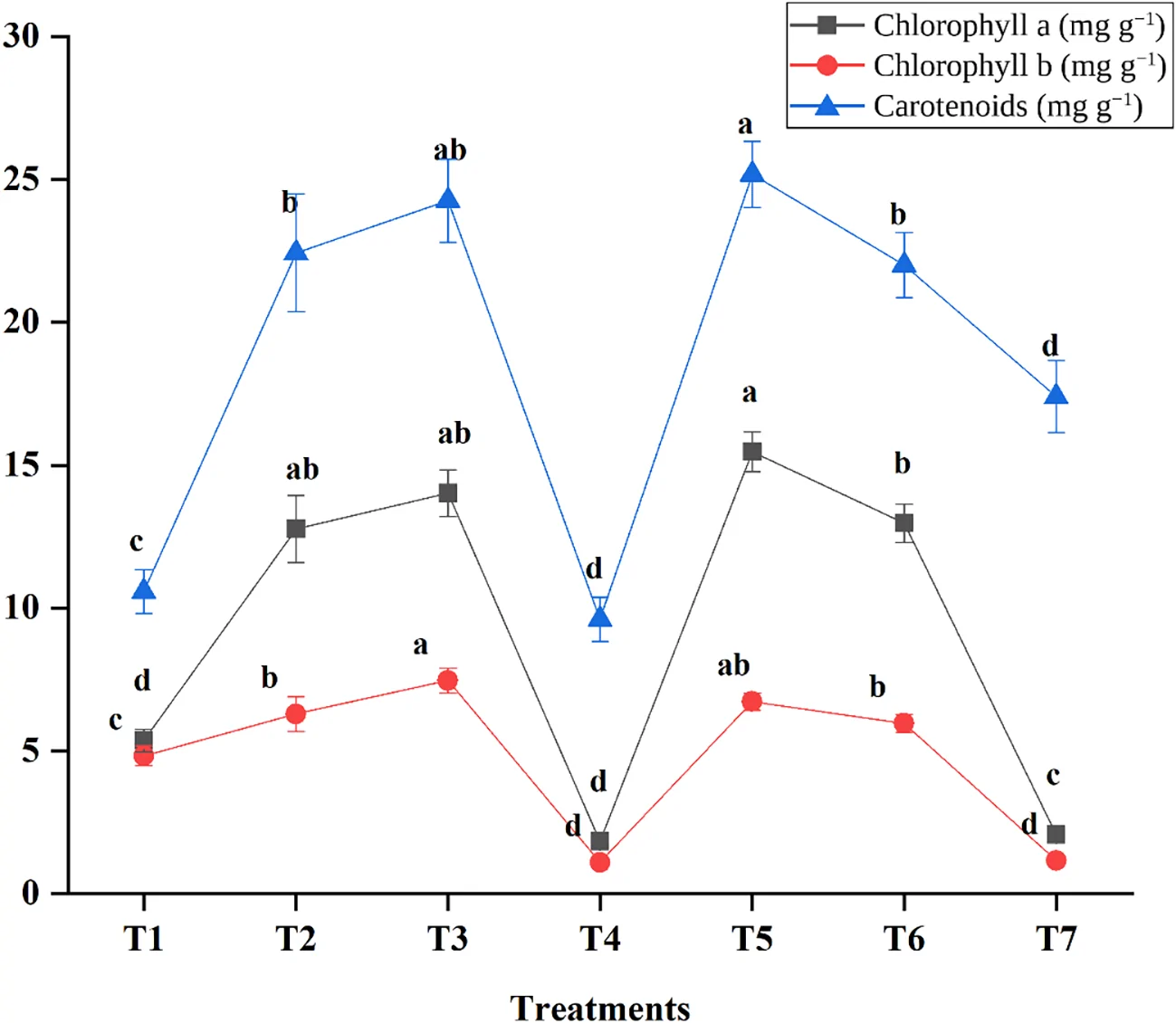
Effects of soil, foliar, and combined soil + foliar selenium application on chlorophyll *a*, chlorophyll *b*, and carotenoid contents in okra (*Abelmoschus esculentus* L.) leaves. T_1_, soil application (25 ppm Se); T_2_, soil application (50 ppm Se); T_3_, foliar application (25 ppm Se); T_4_, foliar application (50 ppm Se); T_5_, combined soil + foliar application (25 ppm Se); T_6_, combined soil + foliar application (50 ppm Se); T_7_, untreated control (deionized water). Values are presented as mean ± standard error (SE) (n = 3). Different lowercase letters above the bars indicate significant differences among treatments according to Fisher’s least significant difference (LSD) test at *P* ≤ 0.05.

**Figure 5:**
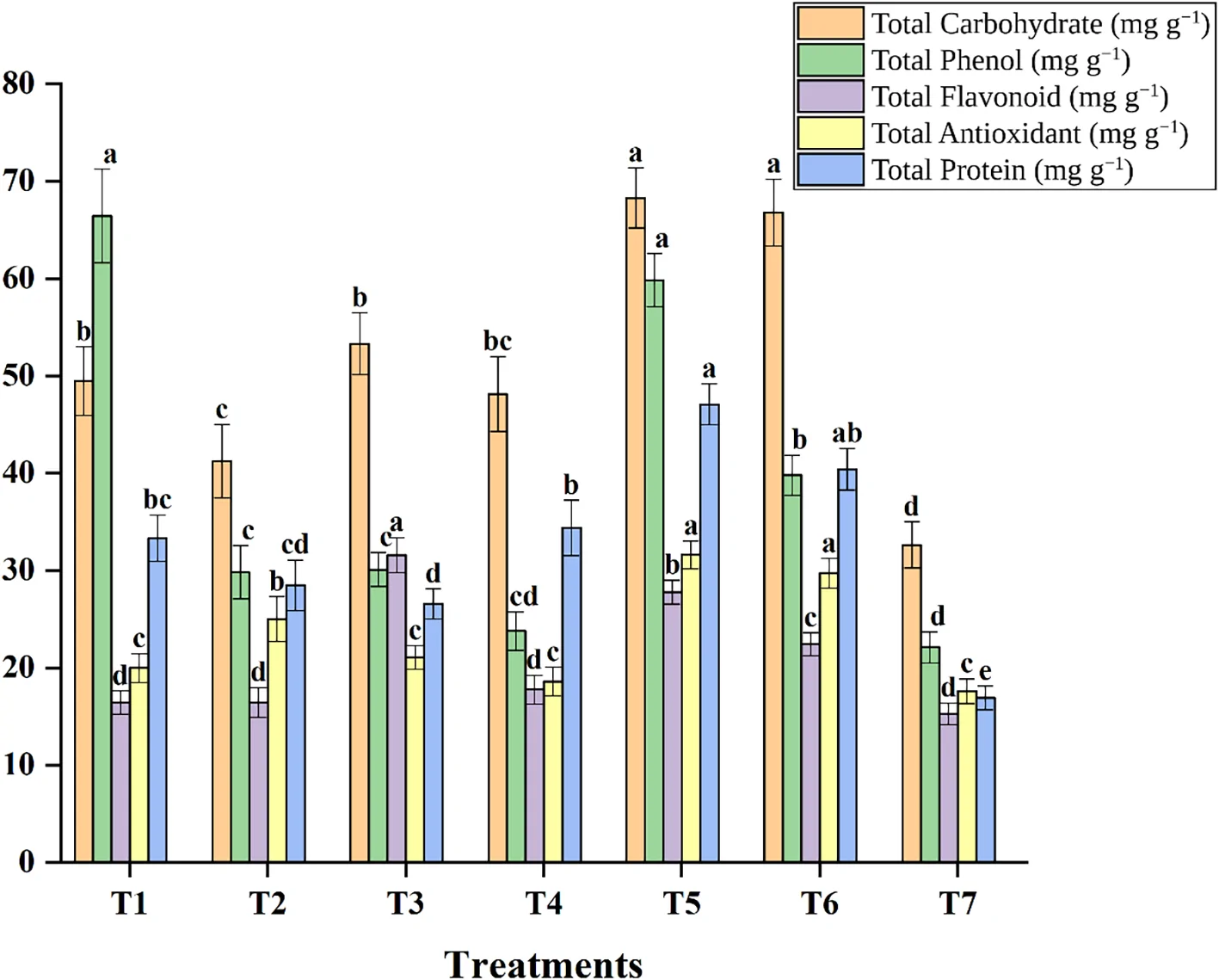
Effects of soil, foliar, and combined soil + foliar selenium application on total carbohydrate, total phenolic, total flavonoid, antioxidant, and total protein contents in okra (*Abelmoschus esculentus* L.). T_1_, soil application (25 ppm Se); T_2_, soil application (50 ppm Se); T_3_, foliar application (25 ppm Se); T_4_, foliar application (50 ppm Se); T_5_, combined soil + foliar application (25 ppm Se); T_6_, combined soil + foliar application (50 ppm Se); T_7_, untreated control (deionized water).Values are presented as mean ± standard error (SE) (n = 3). Different lowercase letters above the bars indicate significant differences among treatments according to Fisher’s least significant difference (LSD) test at *P* ≤ 0.05.

The enhanced chlorophyll content observed in T_5_ may also be related to improved magnesium uptake and increased activity of enzymes involved in chlorophyll biosynthesis. Similar increases in chlorophyll and carotenoid content following selenium treatments have been reported in grapevine, lettuce, rice, and medicinal plants (Dey and Raychaudhuri, 2023; Hawrylak-Nowak et al., 2010). Increased carotenoid accumulation further suggests enhanced photoprotective capacity and antioxidant activity in selenium-treated plants.

Total carbohydrate content was significantly higher in T_5_ (68.27 mg g^-1^) than in the control (32.63 mg g^-1^), reflecting improved carbon fixation and metabolic efficiency. Selenium treatments have previously been shown to stimulate carbohydrate metabolism and improve translocation of photosynthates through enhanced enzymatic regulation and membrane permeability (Ahmed et al., 2020). Enhanced carbohydrate accumulation contributes directly to improved fruit development and yield performance.

Selenium treatment also significantly increased phenolic compounds, flavonoids, antioxidant activity, and protein content. The highest phenolic (59.83 mg g^-1^), flavonoid (27.77 mg g^-1^), antioxidant (31.60 mg g^-1^), and protein contents (47.07 mg g^-1^) were recorded in T_5_. These findings indicate that selenium treatments activated antioxidant defense pathways and secondary metabolite biosynthesis. Selenium-induced accumulation of phenolics and flavonoids has been associated with activation of the phenylpropanoid pathway and enhanced ROS-scavenging capacity (Feng et al., 2013; Aftab, 2024; Zhang et al., 2025). The increased antioxidant activity observed in selenium-treated plants suggests improved tolerance against oxidative stress through regulation of antioxidant enzymes and non-enzymatic antioxidant compounds. Similar findings have been reported in selenium-biofortified leafy vegetables and medicinal crops, where selenium significantly improved antioxidant potential and nutritional quality (Francini et al., 2023; Jam et al., 2026). Enhanced protein accumulation under selenium treatment may also be linked to improved nitrogen metabolism and sulfur assimilation pathways because selenium shows metabolic similarities with sulfur-containing amino acids (Bhadwal and Sharma, 2022).

Collectively, the present findings demonstrate that moderate selenium treatments significantly improve metabolic efficiency, antioxidation defense mechanisms, and nutritional quality in okra. These responses support the role of selenium biofortification as a sustainable agricultural strategy for producing nutrient-enriched vegetables with improved functional and health-promoting properties.

### 3.3 Mineral content

Selenium treatments significantly affected the accumulation of essential macro- and micronutrients in okra pods (**Table 3**). Treatment T_5_ exhibited the highest calcium (17,690 µg g^-1^), magnesium (37,460 µg g^-1^), potassium (58,600 µg g^-1^), and iron contents (10,380 µg g^-1^), where as in the control T_7_ recorded (MDL), indicating that the selenium concentration was below the detectable limit. Selenium was below the method detection limit (MDL) in the pods of the untreated control (T_7_), whereas selenium was detected in the pods of the selenium-treated plants, indicating treatment-associated selenium accumulation. Selenium and iodine contents were also enhanced in selenium-treated plants, indicating that selenium biofortification offers an effective approach for improving the nutritional quality. The improved mineral accumulation observed under T_5_ may be associated with selenium-mediated enhancement of membrane permeability, root activity, ion transport efficiency, and nutrient uptake mechanisms. Selenium has been shown to positively regulate the uptake of essential nutrients such as nitrogen, phosphorus, potassium, calcium, magnesium, zinc, and iron by improving root physiology and transporter activity (Raina et al., 2020). Similar increases in mineral accumulation following selenium treatments have been reported in rice, broccoli, wheat, and leafy vegetables (Broadley et al., 2010; Francini et al., 2023; Wang et al., 2021).

**Table 3:** Effects of soil, foliar, and combined soil + foliar selenium applications on the mineral composition of okra pods (*Abelmoschus esculentus* L.) under open-field conditions.

Treatment	Iodine (µg g^-1^)	Selenium (µg g^-1^)	Calcium (µg g^-1^)	Magnesium (µg g^-1^)	Potassium (µg g^-1^)	Iron (µg g^-1^)
T1	170	9	15,630	31,950	34,410	5,670
T2	150	21	12,910	30,310	29,290	5,840
T3	210	22	11,990	33,020	34,650	3,970
T4	200	68	14,700	31,960	33,200	3,810
T5	250	52	17,690	37,460	58,600	10,380
T6	230	20	17,270	34,740	32,540	7,190
T7	50	MDL	13,060	30,790	31,760	3,170

Values in the table represent a single composite sample per treatment obtained by pooling all field replications.

The combined soil and foliar application strategy likely enhanced nutrient bioavailability and selenium translocation within plant tissues more effectively than individual application methods. Soil application ensured continuous selenium availability for root uptake, whereas foliar application facilitated rapid absorption and redistribution to metabolically active tissues (Li et al., 2025). Similar findings were reported by Ros et al. (2016), who demonstrated that integrated selenium fertilization strategies improve selenium use efficiency and biofortification potential in food crops. Increased iron, calcium, and magnesium accumulation under selenium treatments may contribute to enhanced nutritional and functional quality of okra pods. Such improvements are particularly important in addressing micronutrient deficiencies and hidden hunger, which remain major global nutritional challenges. Selenium biofortification offers a promising strategy for developing nutrient-dense vegetables capable of supporting sustainable food systems and nutritional security. However, selenium concentration must be carefully optimized because excessive selenium accumulation may disrupt nutrient homeostasis and induce phytotoxic effects. Previous studies have demonstrated that high selenium levels may interfere with sulfur metabolism and negatively affect nutrient balance in sensitive crop species (Gui et al., 2021; Liu et al., 2024). Therefore, maintaining selenium within optimal physiological limits is essential for maximizing agronomic and nutritional benefits without compromising plant health.

The present study demonstrates that combined soil and foliar application of selenium at 25 ppm significantly improved growth, yield, biochemical constituents, antioxidant activity, and mineral accumulation in okra under open-field conditions. These findings highlight the potential of selenium-based agronomic biofortification as an environmentally sustainable and nutrition-sensitive agricultural strategy capable of enhancing crop productivity, improving food quality, and supporting future food and nutritional security under changing climatic conditions.

## 4 Conclusion

The present study demonstrated that moderate selenium treatments, particularly through the combined soil and foliar application of 25 ppm Se (T_5_), significantly improved the growth, yield, biochemical composition, and mineral accumulation of okra (*Abelmoschus esculentus* L.) under open-field conditions. Among all treatments, T_5_ consistently showed significant effects by enhancing vegetative growth, flowering, pod development, and overall crop productivity, resulting in an 58% increase in yield compared with the control. Enhanced chlorophyll, carotenoid, carbohydrate, protein, phenolic, flavonoid, and antioxidant contents under T_5_ indicate that selenium plays a vital role in improving photosynthetic efficiency, metabolic activity, nutrient utilization, and oxidative stress tolerance. Selenium application also enhanced the accumulation of essential macro- and micronutrients, thereby improving the nutritional quality and biofortification potential of okra pods.

These findings highlight the importance of selenium-based agronomic biofortification as a sustainable approach for improving crop productivity and nutritional security under changing environmental conditions. In the context of increasing food demand, climate variability, declining soil fertility, and micronutrient malnutrition, selenium-mediated biofortification offers a promising strategy for developing resilient and nutrition-sensitive vegetable production systems. The combined soil and foliar application approach demonstrated in this study supports the integration of micronutrient management with sustainable agricultural practices to produce nutrient-dense crops while maintaining environmental sustainability. Furthermore, selenium-induced enhancement of antioxidant defense mechanisms suggests its potential role in improved growth, yield, and nutritional quality under open-field cultivation and may contribute to sustainable crop production. However, limited information is available regarding the optimization of selenium dosage, application timing, and integrated nutrient management practices across different agroecological conditions. Long-term studies on selenium dynamics, environmental safety, and consumer health impacts are therefore essential for the sustainable adoption of selenium-enriched vegetables and their integration into future sustainable agricultural systems.

## Data Availability

The raw data supporting the conclusions of this article will be made available by the authors, without undue reservation.
